# Supplementary material for: The Molecular Resistance Mechanisms of European Earwigs from Apple Orchards Subjected to Different Management Strategies
Source: Insects. 2023 Dec 13;14(12):944. doi: 10.3390/insects14120944 (PMC10743755; doi:10.3390/insects14120944)
Supplement: Supplementary file 1 [file insects-14-00944-s001.zip › insects-2645801-supplementary.pdf]

Table S1. Primers used in one or two rounds of PCR amplification

Gene category	Gene Name	PCR round	Hybridization temperature (°C)	Forward primer	Reverse primer
Insecticide target genes	Ace 1	1st round	66.5	GTATGAACTCCTGTTGACAAGAAGTGGC	GGAATGCCAGCTTTGGGAAGAGGTG
		2nd round	60.8	TCCTGTTGACAAGAAGTGGCG	ATGCCAGCTTTGGGAAGAGGTG
	Ace 2	1st round	67.5	CGAATATTTCCCAGGCTTCTCCG	ACAAGAGTGGAAAGTGGGAGAAGGATTTGG
	nAChR α1	1st round	53.7	TCAAGCCACAAGCAATCG	TCCATAACGATCCTCTGATC
		2nd round	63.5	CCACAAGCAATCGCCGCATC	CGATCCTCTGATCGCAGACGTC
	nAChR α3	1st round	70	TTCCACCCACAACGCCAACAG	ATTGCCATTGCCATTGCCATTGCC
		2nd round	60	CCACAACGCCAACAGGATCATG	ATTGCCATTGCCATTGCCATTGCC
	nAChR β2	1st round	43	AGATTATTCCAGCTAGATGTC	GAAATGTAGCGAAGGTACGA
		2nd round	69.7	AGATGTCCTGGAGCAACCTGTG	GCGAAGGTACGAATTGAGTTCCG
	GluCl iso1	1st round	57.5	CCACATAGTTCCACCATGCT	ACTTGATAGATGGGTGGGTT
		2nd round	66.5	ACATAGTTCCACCATGCTGTGGTATG	GATGGGTGGGTTTTGAGATCATAGTC
	GluCl iso2	1st round	59.2	CCACATAGTTCCACCATGCT	ACTTGATAGATGGGTGGGTT
		2nd round	63.5	ACATAGTTCCACCATGCTGTGGTATG	GATGGGTGGGTTTTGAGATCATAGTC
	Para F1	1st round	58.5	CCCATAAACCCACAAACCCCTTC	CACCCAGTTTTCATGCGTAAGTTC
		2nd round	63.5	TTGACGTCTTCGACGTCGAGCC	CACCCAGTTTTCATGCGTAAGTTC
	Para F2	1st round	66.5	GGATCATCACGATATGATAAGGGACATGGC	GCTAAGCGTATCTTCAATAATCGAGCCAG

	Para F3	1st round	59.2	GATCTCGAAGAAGAAGAAGGCG	AGCGCGTTTCAGGACGGATC
		2nd round	70	CTCGAAGAAGAAGAAGGCGAAGAGC	GTTTCAGGACGGATCGAGACGG
	RyR	1st round	60.8	GGATGACTCGAATTGCAGCCTATACTC	CAGCATCTTCAAACGATTGAGTGAG
Detoxification genes	CCE B	1st round	59.2	CTGGATCGCTTCGGTTCAAG	GGCAATCCTCGTTTCCATCC
	CCE E	1st round	60	AGCGAAGAAAATCAGCCGTG	CTTCCGATCTGTTTATTTAAGTTTAC
		2nd round	62	GAAGAAAATCAGCCGTGTTCGTAATG	CTGTTTATTTAAGTTTACAATTCGTCAGG
	CYP4G189	1st round	60	GATCATTCAAGTGGCCTCATTGATAAG	CGATTCTATTCCATTGAGCTAGAAGG
		2nd round	66.5	CATTCAAGTGGCCTCATTGATAAG	CTATTCCATTGAGCTAGAAGGAGG
	CYP6NP1	1st round	63.7	ATCATTCCGCCGCATTGAACAGT	CCTGGAATCGAGTCTGGCACTTG
		2nd round	60	CCGCCGCATTGAACAGTTTAACC	GAATCGAGTCTGGCACTTGG
	CYP6NQ1 N-ter	1st round	62	GCACTAACTATGTTACTGTGGATTC	CTCGATTCACTTCTCGATCC
		2nd round	60	GCACTAACTATGTTACTGTGGATTC	GGAGGATACTTCCGCAATGTTTC
	CYP6NQ1 C-ter	1st round	62.5	TCATAGCGTTCTCGAATTGAATGAG	CGTAGGTACCGGATACTGGATAC
	CYP6NW1	1st round	63.5	AACCGCGAGACCAATGGATCTCC	GCGCATGCGCACATTTATCCTTTC
	GST D	1st round	55.6	ACTGCACACGCGTCCATTG	TCCATTTAGAGCACAGAAGTGTAGG
		2nd round	60	GCACACGCGTCCATTGGAATATC	GAGCACAGAAGTGTAGGTATTATTG
Control genes	Actin	1st round	58.5	GCTCTTCCGATCTCCTACAGCAC	GTCTTTCTGCATAAACTTTTGGTGG
	EF1alpha	1st round	66.5	GGGTACAAAGGAGCAATCGGC	GAAAGAGGGAGGAGTCTACAGCTC
	GAPDH	1st round	63.5	CATTAACCTTGCCGTGTCATCAGCC	GTTCTTACAGTGAGTGGGCTTACTGC

Table S2. Primers used in quantitative real-time PCR

Gene Category	Gene Name	Forward Primer	Reverse Primer	Amplicon size (bp)	PCR efficiency (%)
Insecticide target genes	Ace 1	CACGAGGTGGTCAGATGTCT	TGGGGATTCGAGGATCATGG	86	101
	nAChR α1	GGATTTTCACGGTGGCTTGT	CTTCCGGACCCATCATCATC	144	110
	nAChR α3	CGGATGGCAATTTCGAGGTG	CCACGTCTATTTCGCACGAG	111	98
	nAChR β2	CACCTTCCTAACAGTTCTCGTT	GAAAGCAAGATGGACACGCA	81	101
Detoxification genes	CCE B	CTGGATCGCTTCGGTTCAAG	GGCAATCCTCGTTTCCATCC	132	105
	CCE E	ATCCTGTGCCCGCCAAAC	CTGGATTCTTAGGAGTGAACAC	150	105
	CYP4G189	TGGAAACGGCTATGGGAGTT	ATGTGGCGGGTATGGAGAAT	100	96
	CYP6NP1	TCCCTGATCCCGAACGTTTC	ACAATTTCGTGGTCCTTCGC	104	104
	CYP6NQ1	CCTGTTATCACGCTTCGGAA	TTCTGGACCACGCAACAAAA	132	115
	CYP6NW1	CGTACTCCGCCAATTAACGA	CAGATGTGAACGTGGGTGAC	112	104
	GST D	TGCCGATTGACCTGTACTACT	TGCAGTTTAACGTCCAGGGA	85	110
Control genes	Actin	GGCTGTGCTCTCGTTGTATG	AGTCAGATCACGACCAGCAA	148	103
	EF1 alpha	CAGTGAGAGCTTCGTGATGC	CAGTCGGTCGAGTTGAAACC	114	103
	GAPDH	TGGTAGAGGTGCTGCTCAAA	ACAGGTACACGGAAAGCCAT	117	101

Loop A, Loop E			
Forficula auricularia α1	92	WNPEDYGGVDTLHVPSEHIWLPDIVLYNNADGNYEVTIMTKAILHHTGKVVWK**P**PAIYKSFCEIDVEYFPFDEQTCFMKFG	172
Forficula auricularia α1 mut		WNPEDYGGVDTLHVPSEHIWLPDIVLYNNADGNYEVTIMTKAILHHTGKVVWK**H**PAIYKSFCEIDVEYFPFDEQTCFMKFG	
Drosophila melanogaster α1		WNPDDYGGVDTLHVPSEHIWLPDIVLYNNADGNYEVTIMTKAILHHTGKVVWK**P**PAIXKSFCEIDVEYFPFDEQTCFMKFG	
Myzus persicae α1		WEPLEYGGVKELYVPSEHIWLPDIVLYNNADGEYVVTTMTKAVLHHSGKVMWT**P**PAIFKSSCEIDVRYFPFDQQTCFMKFG	
Bombyx mori α1		WNPDDYGGVDTLHVPSEHIWLPDIVLYNNADGNYEVTIMTKAILHHDGKVVWK**P**PAIXKSFCEIDVEYFPFDEQTCFMKFG	
Nilaparvata lugens α1		WNPDEYGGVDTLHVPSEHIWLPDIVLYNNADGNYEVTIMTKAILHHTGKVVWK**P**PAIXKSFCEIDVEYFPFDEQTCFMKFG	
Drosophila melanogaster α6		WNETEYGGVKDLRITPNKLWKPDVLMYNSADEGFDGTYHTNIVVKHNGSCLYV**P**PGIFKSTCKIDITWFPFDDQHCEMKFG	
Apis mellifera α6		WNESEYGGVKDLRITPNKLWKPDILMYNSADEGFDGTYQTNVVVTHNGSCLYV**P**PGIFKSTCKIDITWFPFDDQHCDMKFG	
Homo sapiens α6		WDPMEYDGIETLRVPADKIWKPDIVLYNNAVGDFQVEGKTKALLKYNGMITWT**P**PAIFKSSCPMDITFFPFDHQNCSLKFG	

Loop A, Loop E			
Forficula auricularia β2	95	WDPEEYG**G**VEMLYVPSEHIWLPDIVLYNNADGNYEVTLMTKATLKYNGEVFWKPPAIYKSSCRINVEYFPFDEQSCMMKFG	175
Forficula Auricularia β2 mut		WDPEEYG**R**VEMLYVPSEHIWLPDIVLYNNADGNYEVTLMTKATLKYNGEVFWKPPAIYKSSCRINVEYFPFDEQSCMMKFG	
Drosophila melanogaster β2		WDPEEYG**G**VEQLYVPSEHIWVPDIVLYNNWDGNYEVTLMTKATLKYTGEVFWEPPAIYKSSCEMNVEYFPYDEQICFMKFG	
Diabrotica virgifera v. β2		WDPEEYG**G**VEMLYVPSEHIWLPDIVLFNNADGNYEVTLMTKATLSYTGEVIWKPPSIYKSSCEINVQYFPFDEQSCLMKFG	
Homo sapiens β2		WKPEEFDNMKKVRLPSKHIWLPDVVLYNNADGMYEVSFYSNAVVSYDGSIFWLPPAIYKSACKIEVKHFPFDQQNCTMKFR	

Figure S1: Multiple sequence alignments of part of the N-terminal domain containing loops A and E of insects and human nAChR subunits. The two cysteines of the Cys-loop motif ($CX_{13}C$) are highlighted in grey, the position of Faα1 P145H and Faβ2 G102R are in bold. Sequences were retrieved from Genbank Dmα1 NP_524481, Mpα1 CAA57476, Bmα1 ABV45511, Nlα1 BBE49554, Dmα6 NP_723494, Apα6 NP_001073564, Hsα6 CAD88994, Dmβ2 NP_524483, Dvvβ2 XP028137095, Hsβ2 AAD45422
